# The Diagnostic Yields and Safety of Double-Balloon Enteroscopy in Obscure Gastrointestinal Bleeding and Incomplete Small Bowel Obstruction: Comparison between the Adults and Elderly

**DOI:** 10.1155/2020/8121625

**Published:** 2020-04-27

**Authors:** Lei Wang, Mengfan Xie, Liwen Hong, Chen Zhang, Tianyu Zhang, Rong Fan, Jie Zhong, Zhengting Wang

**Affiliations:** Department of Gastroenterology, Ruijin Hospital, Shanghai, Jiaotong University School of Medicine, Shanghai, China

## Abstract

**Background:**

Double-balloon enteroscopy (DBE) is widely used worldwide. However, comparisons between the diagnostic yields in adults and the elderly remain scarce.

**Aim:**

The aim of this study is to compare the diagnostic yields and safety of DBE between adults and elderly with obscure gastrointestinal bleeding and incomplete small bowel obstruction.

**Method:**

We retrospectively reviewed patients who underwent DBE with indication of obscure gastrointestinal bleeding or incomplete small bowel obstruction in Ruijin Hospital and classified them into adults (18–64 years old) and elderly (≥65 years old). Clinical characteristics, diagnostic yields, and postoperative complications were collected and further analyzed.

**Results:**

A total of 877 DBE procedures, 729 in adults and 148 in the elderly, were performed. In the patients with OGIB, the adults showed a higher frequency of Meckel's diverticulum compared with the elderly (4.6% vs. 0.9%, *P* = 0.032). Angioectasia was higher in frequency in the elderly than in the adults (25.9% vs. 17.9%, *P* = 0.048). In patients with incomplete small bowel obstruction, the elderly were more likely to have adenocarcinoma than the adults (19.4% vs. 7.1%, *P* = 0.038). The adults had higher tendency to have Crohn's disease than the elderly (23.4% vs. 8.3%, *P* = 0.045). Most of the postoperative complications were mild. The adults and elderly displayed comparable tolerance to DBE (*P* > 0.05)

**Conclusion:**

DBE has a high diagnostic yield in small bowel disorders, and a slight difference in disease spectrum was observed between the adults and elderly. DBE can be well-tolerated in the elderly.

## 1. Introduction

Double-balloon enteroscopy (DBE) was first introduced by Yamamoto et al. in 2001 [1]. Since then, DBE has revolutionized the clinical approach in diagnosing, sampling, and treating small bowel diseases. Obscure gastrointestinal bleeding (OGIB), which has 69.8%–80.6% diagnostic yield, remains the most common indication for DBE [2, 3]. Other indications for DBE include incomplete small bowel obstruction, abdominal pain, diarrhea, small bowel tumors, and pancreaticobiliary disorders in patients with surgically altered anatomy [4–6]. We previously demonstrated that the indications of OGIB and incomplete small bowel obstruction have the highest diagnostic yields [3]. However, despite its usefulness, DBE is limited by some challenges. Compared with esophagogastroduodenoscopy (EGD), colonoscopy, and video capsule endoscopy (VCE), DBE is more technically complex and requires prolonged duration for endoscope insertion deep into the intestine, and thus, its invasiveness usually increases [7–9]. Therefore, DBE may be recommended to be feasible followed by conventional EGD, colonoscopy, VCE, and CT enterography [10].

Despite its long duration, DBE is a relatively safe procedure with a complication rate comparable to that of conventional endoscopic procedures [11–13]. However, clinicians are disinclined to use DBE in the elderly because it potentially increases risks associated with sedation, comorbidities, and relatively worsened cardiopulmonary function. Data on the use of DBE in elderly and between adults and elderly are limited [14–16]. In this study, we retrospectively compared the diagnostic yields and tolerance between adults and elderly patients who underwent DBE in our center.

## 2. Method

### 2.1. Patients Enrolled

A single-center retrospective study was designed. The medical records of ≥18-year-old patients who underwent DBE with the indication of OGIB or incomplete small bowel obstruction from March 2008 to July 2019 at the Department of Gastroenterology, Ruijin Hospital, Shanghai, China, were reviewed. All of the enrolled patients were divided into two groups by their age (adults: 18–64 years old; elderly: ≥65 years old). Patients with comorbidities of mild-to-moderate chronic diseases, such as hypertension and diabetes mellitus, were included in the analysis. Patients with OGIB had confirmed GI bleeding that is with a symptom of hematochezia or melena or lower hemoglobin plus occult blood test positive with exclusion of non-GI disease. Moreover, they had undergone upper or lower endoscopy and gained no positive findings. This study was approved by the Institutional Ethics Board of Ruijin Hospital.

### 2.2. Preoperative Preparation and DBE Procedure

Written informed consents were acquired from all patients preoperatively. The patients were required to intake a liquid diet with low residue at least 3 days from the procedure. Bowel cleansing was conducted the day before DBE procedure by using at least 3.0 L of a compound laxative agent with 132 g of polyethyleneglycol as the major component.

DBE (EN-450 P5/20, Fujifilm, China) was performed by three endoscopists (JZ, SC, and LW). The DBE system consists of a 200 cm long endoscope with a 140 cm overtube. The endoscope and overtube were equipped with an inflatable balloon at their distal tips. The bowel could be anchored through alternatively inflating the balloon of the endoscope or overtube. Thus, the endoscope could be inserted deep into the intestine.

The route of DBE procedure (antegrade or retrograde) was determined under the guidance of clinical symptoms and diagnostic tools. Patients who underwent both routes of the procedure were regarded to have underwent two separate DBEs. Conscious sedation with midazolam and fentanyl was induced in some patients during the DBE procedure.

### 2.3. Data Collection

Patients' gender, duration of symptom, past GI surgery, DBE time, route, and length of the examined intestine were recorded as clinical characteristics. The diagnostic yields of DBE with the indication of OGIB, including normal, adenocarcinoma, lymphoma, GISTs, Meckel's diverticulum, polyps, Crohn's disease, intestinal tuberculosis, Behcet's disease, angioectasia, and nonspecific enteritis, were retrieved. The diagnostic yields of DBE with the indication of incomplete small bowel obstruction, including normal, adenocarcinoma, lymphoma, GISTs, Crohn's disease, intestinal tuberculosis, Behcet's disease, and cryptogenic multifocal ulcerous stenosing enteritis (CMUSE), were also analyzed. Finally, the complications of DBE, including abdominal pain, abdominal distension, diarrhea, nausea, perforation, and bleeding, were compared between the adults and elderly.

### 2.4. Statistical Analysis

SPSS 19.0 was used for data analyses. Continuous variables were expressed as mean ± SD. Student's *t-*test and Wilcoxon rank sum test were performed for data comparison with and without normal distribution, respectively. A probability (*P*) value of <0.05 was considered statistically significant.

## 3. Results

### 3.1. Clinical Characteristics of the Adults and Elderly Who Underwent DBE

During the study period, 787 patients were enrolled, 88.6% (697/787) of which received one route of DBE, and 11.4% (90/787) underwent both routes. A total of 877 DBEs were performed, 729 in the adults and 148 in the elderly. The clinical characteristics of the adults and elderly who underwent DBE are displayed in Table 1. No significant difference in gender and the indications of OGIB and incomplete small bowel obstruction was found between the adults and elderly (*P* > 0.05). In the patients who underwent DBEs, the mean duration of symptoms was approximately 12 weeks (12.22 ± 2.89 and 12.24 ± 3.10 weeks, *P* = 0.944). The elderly were more likely to have past GI surgery than the adults; however, no significant difference was found (*P* = 0.161). In the parameters of DBE maneuvering, such as DBE time, route, and examined intestine length, no significant difference was observed between the adults and elderly (*P* > 0.05).

### 3.2. Diagnostic Yields of DBE between the Adults and Elderly with OGIB

The diagnostic yields of DBE between the adults and elderly with OGIB are listed in Table 2. Among the patients who underwent DBE, approximately 14.3%–15.1% were normal in both groups (*P* = 0.817). The adults had higher tendency to have Meckel's diverticulum than the elderly (4.6% vs. 0.9%, *P* = 0.032). On the contrary, the elderly were more likely to have angioectasia than the adults (25.9% vs. 17.9%, *P* = 0.048). The two groups were comparable in diagnostic findings, such as adenocarcinoma, lymphoma, GISTs, polyps, Crohn's disease, intestinal tuberculosis, Behcet's disease, and nonspecific enteritis (*P* > 0.05).

### 3.3. Diagnostic Yields of DBE between the Adults and Elderly with Incomplete Small Bowel Obstruction

The diagnostic yields of DBE between the adults and elderly with incomplete small bowel obstruction are displayed in Table 3. The negative ratios were approximately 11.1%–12.8%. The elderly showed a higher frequency of adenocarcinoma than the adults (19.4% vs. 7.1%, *P* = 0.038). However, the adults had a higher frequency of Crohn's disease than the elderly (23.4% vs. 8.3%, *P* = 0.045). In terms of lymphoma, GISTs, intestinal tuberculosis, Behcet's disease, and CMUSE, both groups showed comparable ratios (*P* > 0.05).

### 3.4. Postoperative Complications of DBE in the Adults and Elderly

The postoperative complications of DBE in the adults and elderly are shown in Table 4. The main postoperative complications of DBE were mild and included abdominal pain, abdominal distension, diarrhea, and nausea. The adults and elderly displayed comparable complications (*P* > 0.05). Postoperative bleeding occurred in two patients, with no significant difference between the adults and elderly (*P* = 0.426). Only 1 patient in both groups suffered from perforation, a severe complication (*P* = 0.169).

## 4. Discussions

The advent of the aging society in China has brought great concerns on elderly health care. Elderly patients have distinct disease spectrum and well-being. The diagnostic yield and tolerability of DBE in elderly patients compared with adults should be determined.

Given its technical complexity and potential invasiveness, DBE is not always the first choice in detecting small bowel lesions. The diagnostic efficacy and yield of DBE have become a major concern for physicians. In our previous findings, OGIB and incomplete small bowel obstruction have the highest (approximately 80%) diagnostic yields compared with other indications [3]. This finding prompted us to further discover the discordance of diagnostic yields between adults and elderly. This study retrospectively reviewed 877 DBEs within 10 years in our hospital. We believe that this population is a fairly large cohort that may represent the characteristics and outcomes of Chinese adults and elderly patients receiving DBEs.

This study showed that over 10 years, the overall diagnostic yields of DBE in OGIB or incomplete small bowel obstruction had increased to 84.9%–88.9%, which is much higher than our previous findings [3] and those of a recent study in China by Wang et al. [17]. This result might be ascribed to the optimization of DBE indication and the improved guidance of other diagnostic modalities, such as CT enterography and MR enterography. For patients with OGIB, Meckel's diverticulum was more observed in the adults than in the elderly and the reverse in angioectasia. These findings further indicated that the proportion of lesions in adults and elderly had some difference. In patients with incomplete small bowel obstruction, Crohn's disease was more prevalent in the adults than in the elderly, which may be due to the high incidence of Crohn's disease in young patients [18]. On the contrary, the elderly had a higher incidence of adenocarcinoma than the adults. This finding might be ascribed to the high chance of malignancy in the elderly [19]. Our findings were similar to those of other studies [13, 15, 20, 21].

Despite the usefulness of DBE in diagnosing small bowel disease, its safety should be analyzed in the elderly. Furthermore, approximately 12.0%–20.4% of the patients underwent discomfort postoperatively. Most of the discomforts were mild and could be successfully alleviated through symptomatic treatment. The complication rate was higher than that observed in other researches [12–14, 16]. A possible reason is that many patients did not receive midazolam and fentanyl for sedation, for they had to change body positions frequently to facilitate deeper insertion. These medicines are generally administered to improve patient cooperation and ameliorate discomfort. No significant difference in complication was found between the adults and elderly, further proving the safety of DBE in the elderly. Only one patient experienced perforation after the procedure, which might be ascribed to the multiple operations and colostomy he received. This finding suggests the careful performance of DBE in patients with postoperative ankylenteron or surgically altered anatomy.

Our study has several limitations. First, this study was designed retrospectively. Second, we defined elderly as individuals aged ≥65 years and did not subdivide the elderly into additional groups for evaluation. In the future, we could carry out additional prospective studies with much old patients enrolled to further analyze the feasibility and safety of DBE in the extremely old population.

In conclusion, DBE has high a diagnostic yield in small bowel disorders with slightly different disease spectrum between the adults and elderly. Most of the postoperative complications of DBE is mild and could be properly alleviated. The tolerance of the elderly to DBE is comparable to that of the adults.

## Figures and Tables

**Table 1:** Clinical characteristics of adult and elderly patients underwent DBE.

	Adults (*n* (%))	Elderly (*n* (%))	*P*
Total	729	148	/
Gender (M/F)	401/328	82/66	0.929
Indications			0.169
Obscure gastrointestinal bleeding	588 (80.7)	112 (75.7)	
Incomplete small bowel obstruction	141 (19.3)	36 (24.3)	
Duration of symptom (week)	12.22 ± 2.89	12.24 ± 3.10	0.944
Past GI surgery	58 (9.9)	17 (11.5)	0.161
DBE time (min)	66.49 ± 26.10	64.43 ± 25.37	0.335
DBE route			0.819
Antegrade	298 (40.9)	62 (41.9)	
Retrograde	431 (59.1)	86 (58.1)	
Length of examined intestine (cm)	130.67 ± 36.38	129.48 ± 37.40	0.691

**Table 2:** Diagnostic yields of DBE between adults and elderly patients with OGIB.

	Adults N = 588 (*n* (%))	Elderly N = 112 (*n* (%))	*P*
Normal	89 (15.1)	16 (14.3)	0.817
Adenocarcinoma	33 (5.6)	8 (7.1)	0.527
Lymphoma	37 (6.3)	7 (6.3)	0.986
GISTs	52 (8.8)	9 (8.0)	0.781
Meckel's diverticulum	27 (4.6)	1 (0.9)	0.032
Polyps	76 (12.9)	15 (13.4)	0.893
Crohn's disease	76 (12.9)	11 (9.8)	0.361
Intestinal tuberculosis	28 (4.8)	4 (3.6)	0.580
Behcet's disease	27 (4.6)	5 (4.5)	0.953
Angioectasia	105 (17.9)	29 (25.9)	0.048
Nonspecific enteritis	27 (4.6)	4 (3.6)	0.736
Others	11 (1.9)	3 (2.7)	0.621

**Table 3:** Diagnostic yields of DBE between adults and elderly patients with incomplete small bowel obstruction.

	Adults N = 141 (*n* (%))	Elderly N = 36 (*n* (%))	*P*
Normal	18 (12.8)	4 (11.1)	0.786
Adenocarcinoma	10 (7.1)	7 (19.4)	0.038
Lymphoma	15 (10.6)	3 (8.3)	0.676
GISTs	9 (6.4)	3 (8.3)	0.685
Crohn's disease	33 (23.4)	3 (8.3)	0.045
Intestinal tuberculosis	15 (10.6)	6 (16.7)	0.336
Behcet's disease	13 (9.2)	4 (11.1)	0.735
CMUSE	22 (15.6)	4 (11.1)	0.497
Others	6 (4.3)	2 (5.6)	0.744

**Table 4:** Postoperative complications of DBE in adults and elderly patients.

	Adults (*n* (%))	Elderly (*n* (%))	*P*
Abdominal pain	19 (2.6)	6 (4.1)	0.357
Abdominal distention	17 (2.3)	6 (4.1)	0.260
Diarrhea	22 (3.0)	4 (6.8)	0.835
Nausea	28 (3.8)	7 (4.7)	0.615
Bleeding	2 (0.3)	0 (0.0)	0.426
Perforation	0 (0.0)	1 (0.7)	0.169

## Data Availability

Data was available through contacting Prof. Jie Zhong using email.
